# Plasma Soluble ST2 as a Prognostic Biomarker for Cardiovascular Events and Mortality in COVID-19 Patients

**DOI:** 10.3390/jcdd12070273

**Published:** 2025-07-17

**Authors:** Yongcui Yan, Yan Zhuang, Huihui Li, Dao Wen Wang

**Affiliations:** Division of Cardiology and Hubei Key Laboratory of Genetics and Molecular Mechanisms of Cardiological Disorders, Tongji Hospital, Tongji Medical College, Huazhong University of Science and Technology, Wuhan 430030, China; yongcui_yan@163.com (Y.Y.); zy2358292726@163.com (Y.Z.); whimsyhui@163.com (H.L.)

**Keywords:** COVID-19, soluble ST2, cardiovascular events, prognosis

## Abstract

Background: Coronavirus disease 2019 (COVID-19) is frequently complicated by cardiovascular involvement. Soluble growth stimulation-expressed gene 2 (sST2) is a promising cardiovascular biomarker, but its prognostic value in COVID-19 remains unclear. Methods: This retrospective cohort study included 314 hospitalized COVID-19 patients classified into mild/moderate (*n* = 168) and severe/critical (*n* = 146). Plasma sST2 were measured using an enzyme-linked immunosorbent assay. Correlation analyses evaluated associations between sST2 and clinical parameters. Cox regression assessed the independent predictive value for cardiovascular events and all-cause mortality. Results: sST2 levels were significantly higher in severe/critical patients (16.877 ng/mL) than in mild/moderate cases (6.189 ng/mL) and healthy controls (4.003 ng/mL). sST2 positively correlated with cardiac injury markers (cTnI, CK-Mb, NT-proBNP), inflammatory indices (IL-1β, hsCRP), D-dimer, and inversely correlated with a left ventricular ejection fraction (r = −0.86). Elevated sST2 independently predicted cardiovascular events (HR = 2.972) and mortality (HR = 4.681). The Kaplan–Meier survival analysis demonstrated higher cardiovascular event rates and lower survival probabilities in patients with elevated sST2. The ROC curve indicated sST2 outperformed cTnI and NT-proBNP in predicting cardiovascular events (AUC = 0.898) and mortality (AUC = 0.871). Conclusion: Elevated sST2 is associated with myocardial injury, inflammation, and poor prognosis in COVID-19, supporting its value for risk stratification.

## 1. Introduction

Coronavirus disease 2019 (COVID-19), caused by severe acute respiratory syndrome coronavirus 2 (SARS-CoV-2), has precipitated a global health crisis with widespread systemic effects, including the cardiovascular system [1,2]. Emerging evidence indicates that COVID-19 is associated with a heightened risk of cardiovascular complications, including acute myocardial injury, myocarditis, various arrhythmias, microvascular angiopathy, left ventricular dysfunction, heart failure, acute coronary syndrome, and acute pericarditis, all of which contribute to increased morbidity and mortality among hospitalized patients [3,4]. The pathophysiology underlying COVID-19-related cardiovascular injury is multifaceted, involving the direct viral invasion of myocardial tissue, endothelial dysfunction, systemic inflammation, and hypercoagulability [5]. Given the substantial burden of cardiovascular complications, early identification of high-risk COVID-19 patients is crucial for optimizing clinical management and improving outcomes.

Soluble growth stimulation-expressed gene 2 (sST2), a member of the interleukin-1 receptor family, has emerged as a promising biomarker for cardiac stress, myocardial fibrosis, and systemic inflammation [6,7]. Previous studies have demonstrated the diagnostic and prognostic significance of sST2 across various cardiovascular conditions. In heart failure, elevated sST2 concentrations have been correlated with increased mortality and rehospitalization rates, independent of traditional biomarkers such as B-type natriuretic peptide (BNP) and N-terminal pro-BNP (NT-proBNP) [8,9]. Similarly, in acute coronary syndromes, higher sST2 levels have been associated with a greater risk of adverse outcomes, including recurrent myocardial infarction and cardiovascular death [10,11]. Furthermore, our previous study identified sST2 as a sensitive and specific biomarker for fulminant myocarditis, underscoring its role as a key indicator of myocardial stress and systemic inflammation [12]. Given that COVID-19 induces a hyperinflammatory state, endothelial injury, and myocardial stress, it is plausible that sST2 levels may provide critical insights into disease severity, cardiovascular risk, and patient prognosis. However, data regarding the prognostic value of sST2 in cardiovascular risks associated with COVID-19 remain limited.

In this study, we investigate the clinical significance of plasma sST2 levels in hospitalized COVID-19 patients by evaluating its association with myocardial injury, inflammation, and coagulation dysfunction. Furthermore, we assess the prognostic value of sST2 in predicting cardiovascular events and all-cause mortality and compare its diagnostic performance with conventional cardiac biomarkers and inflammatory cytokines. Our findings identify sST2 as a superior prognostic biomarker, offering greater predictive accuracy for adverse cardiovascular outcomes in COVID-19 patients.

## 2. Methods

### 2.1. Study Population

This retrospective cohort study enrolled 314 hospitalized COVID-19 patients who were admitted to Tongji Hospital, Wuhan, China, between January 2023 and March 2023. In addition, 213 healthy controls without a history of COVID-19 infection were included for comparison. The study was approved by Tongji Hospital and Tongji Medical College ethical review board (ID: TJ- IRB20210138) and was conducted in accordance with the principles outlined in the Declaration of Helsinki. Written consent was obtained from all participants after being fully informed.

Patients were eligible for inclusion if they met the following criteria: (1) age ≥ 18 years; (2) laboratory-confirmed SARS-CoV-2 infection via polymerase chain reaction (PCR) testing of respiratory specimens or serological evidence (IgM/IgG) with compatible respiratory symptoms; and (3) complete clinical data. Exclusion criteria included the following: (1) history of malignancy, end-stage renal disease requiring dialysis, or liver cirrhosis; (2) acute myocardial infarction (AMI) or acute coronary syndrome within 30 days before admission; (3) pre-existing severe chronic heart failure (left ventricular ejection fraction (LVEF) < 40%) unrelated to COVID-19; (4) documented acute myocarditis of non-COVID-19 origin (e.g., autoimmune myocarditis, biopsy-confirmed viral myocarditis unrelated to SARS-CoV-2 infection); and (5) patients with incomplete medical records or missing critical laboratory data.

In our study, patients were classified into two groups based on the World Health Organization (WHO) COVID-19 severity classification criteria [13]: mild/moderate group (*n* = 168); severe/critical group (*n* = 146). Severe/critical illness was defined by the presence of at least one of the following criteria: (1) severe respiratory distress with a respiratory rate ≥30 breaths/min; (2) oxygen saturation (SpO_2_) ≤ 93% while breathing ambient air at rest; (3) PaO_2_/FiO_2_ ratio ≤ 300 mmHg; (4) radiologic evidence of rapid pulmonary lesion progression (>50% within 24–48 h); (5) respiratory failure requiring mechanical ventilation; (6) shock; and (7) other organ failure necessitating intensive care unit admission.

### 2.2. Data Collection and Laboratory Assessments

Demographic characteristics, clinical symptoms, comorbidities, laboratory parameters, imaging findings, and in-hospital outcomes were extracted from electronic medical records at the time of admission. The collected data included age, sex, clinical symptoms (fever, cough, sputum production, shortness of breath, dyspnea, chest pain, chest distress, fatigue, and gastrointestinal symptoms), and comorbidities (hypertension, diabetes, hyperlipidemia, chronic renal failure, chronic liver disease, chronic lung disease, history of solid organ transplantation, and obesity [BMI ≥ 30 kg/m^2^]). Additional laboratory analyses included the quantification of cardiac biomarkers (cTnI, CK-Mb, Mb, and NT-proBNP), inflammatory cytokines (IL-10, IL-1β, IL-2, IL-2R, IL-4, and hsCRP), and hematological and biochemical parameters (WBC, lymphocytes, neutrophils, platelets, hemoglobin, LDL, HDL, ALT, AST, creatinine, LDH, PaCO_2_, and PaO_2_). Echocardiographic assessments were conducted by certified cardiologists, with LVEF measured using two-dimensional transthoracic echocardiography following standard protocols. Serum cTnI and CK-Mb were measured using a chemiluminescent immunoassay on the Abbott ARCHITECT platform (Abbott Laboratories, Abbott Park, IL, USA), and plasma NT-proBNP was measured using the Roche Elecsys electrochemiluminescence platform (Roche Diagnostics, Rotkreuz, Switzerland). The 99th-percentile upper reference limit for the hs-cTnI assay is ~34 ng/L in healthy males and ~16 ng/L in females, and for NT-proBNP it is 125 ng/L for ages < 75 and 450 ng/L for ≥75 [14]. All assays were performed in the hospital’s clinical laboratory with standard quality controls.

### 2.3. Measurement of Plasma sST2 Levels

Blood samples were obtained via venipuncture within 48 h of hospital admission and processed by centrifugation at 3000× *g* for 8 min at room temperature to obtain plasma. Plasma sST2 concentrations were measured using a commercially available sandwich enzyme-linked immunosorbent assay (ELISA) kit (Cat#: RK04514, ABclonal Technology, Wuhan, China), which has demonstrated strong correlation (r = 0.81–0.98) with the FDA-cleared Presage^®^ ST2 assay in prior validation studies [15]. The assay has a reported sensitivity of 18.75 pg/mL, with intra-assay and inter-assay coefficients with a variation of <8% and <10%, respectively, at mid-range sST2 concentrations (~15 ng/mL) (https://img.abclonal.com/abclonal-manage/Datasheet/ELISA/RK00377.pdf#:~:text=match%20at%20L435%20CV%28,5, accessed on 2 July 2025). This assay reports sST2 values in ng/mL, consistent with the widely validated and clinically approved Presage^®^ ST2 assay, which is commonly reported in ng/mL units in clinical practice and the published literature [15]. Standard curves were generated for each assay plate using recombinant human sST2 standards provided by the manufacturer. All samples were measured in duplicate, and laboratory personnel were blinded to clinical outcomes.

### 2.4. Study Endpoints and Outcome Definitions

The primary endpoints of this study were cardiovascular events and all-cause mortality during hospitalization. Cardiovascular events were defined as a composite outcome, including cardiovascular death, non-fatal myocardial infarction, non-fatal stroke, and hospitalization due to heart failure. Respiratory failure, shock, and multi-organ failure were recorded as secondary outcomes. Stroke was diagnosed based on the presence of a new-onset, persistent neurological deficit of vascular origin lasting >24 h, confirmed by neuroimaging. Heart failure was diagnosed according to the European Society of Cardiology (ESC) guidelines, requiring typical clinical symptoms, physical examination findings, and objective evidence of cardiac dysfunction.

### 2.5. Statistical Analysis

All statistical analyses were conducted using SPSS (version 26.0, IBM, Armonk, NY, USA), GraphPad Prism (version 10.0), and R software (version 4.3.2, R Project for Statistical Computing). The Kolmogorov–Smirnov test was used to evaluate the normality of the distributions. Continuous variables were presented as medians with interquartile ranges (IQRs). For two-group comparisons, the Mann–Whitney *U* test was used, while comparisons among three groups were performed using the Kruskal–Wallis test. Categorical variables were expressed as counts and percentages and analyzed using the Chi-square test or Fisher’s exact test, as appropriate. The association between sST2 levels and clinical parameters was assessed using Spearman correlation coefficients. The strength and direction of correlation were determined, with statistical significance set at *p* < 0.05. Survival probability was estimated using the Kaplan–Meier method, with patients stratified into high and low sST2 groups based on the median sST2 value. The log-rank test was used to compare survival distributions between groups.

Cox proportional hazards regression models were employed to identify independent predictors of cardiovascular events and all-cause mortality. Univariate Cox regression analysis was first performed to identify potential risk factors. Variables achieving *p* < 0.05 in univariate analysis were included in multivariate models to control for confounding effects. Results were reported as hazard ratios (HRs) with 95% confidence intervals (CIs). The receiver operating characteristic (ROC) curve analysis was conducted to assess the diagnostic performance of sST2 in predicting cardiovascular events and all-cause mortality. The area under the ROC curve (AUC) was calculated for sST2, NT-proBNP, cTnI, IL-1β, and IL-2R. The optimal cutoff values for sST2 were determined using Youden’s index to maximize sensitivity and specificity. All statistical tests were two-sided, and a *p*-value < 0.05 was considered statistically significant.

## 3. Results

### 3.1. Baseline Characteristics of COVID-19 Patients

**Figure 1:**
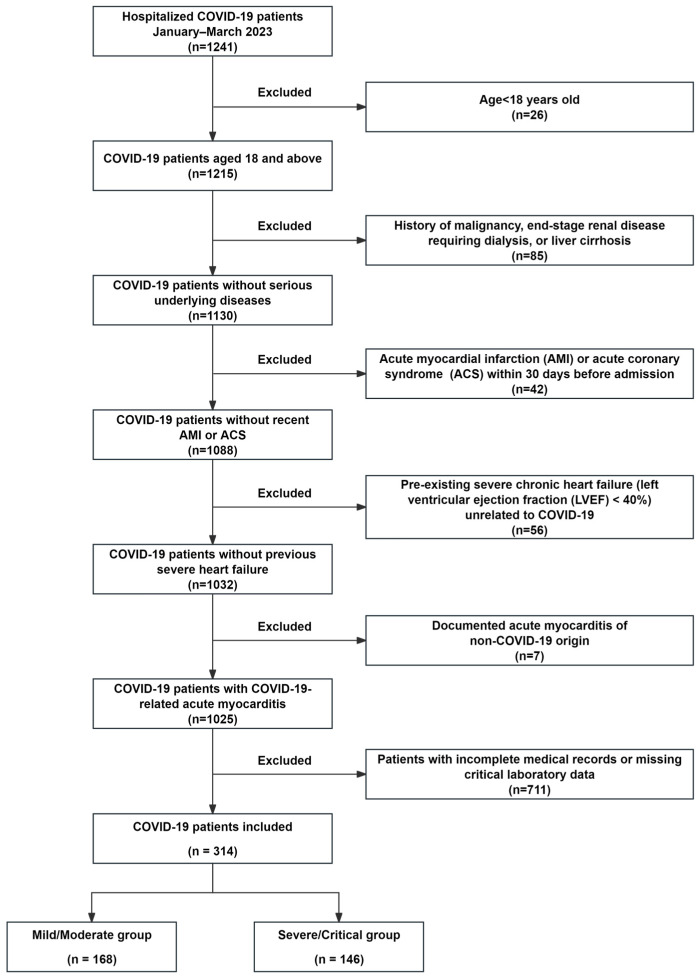
COVID-19 patient enrollment and selection flowchart.

A total of 314 hospitalized COVID-19 patients were enrolled in this study, of whom 168 (53.5%) were classified as mild/moderate cases and 146 (46.5%) as severe/critical cases, based on clinical severity criteria (Figure 1). In addition, 213 healthy controls were included for comparison. The control group had a median age significantly lower than that of the COVID-19 patients (median ~36 years) and a balanced gender distribution (46% male). The demographic and clinical characteristics of both groups are presented in Table 1. Compared to the mild/moderate group, patients in the severe/critical group were significantly older (*p* < 0.001) and predominantly male (*p* = 0.032). In addition, cardiac injury biomarkers, including cTnI, CK-Mb, Mb, and NT-proBNP, were significantly higher in the severe/critical group (*p* < 0.001), suggesting a greater degree of myocardial damage. Echocardiographic evaluation revealed a significant reduction in LVEF in severe/critical cases compared to mild/moderate cases (*p* = 0.023). Moreover, inflammatory cytokines (IL-1β, IL-2R) were significantly elevated in the severe/critical cohort (*p* < 0.05), reflecting a more pronounced inflammatory response. The incidence of respiratory failure, shock, multiple organ failure, and cardiovascular events was also significantly greater in the severe/critical group (*p* < 0.001).

The flowchart illustrates the enrollment and selection of hospitalized COVID-19 patients in this retrospective cohort study. A total of 1241 hospitalized patients were initially screened. Patients were excluded for age < 18 years (*n* = 26), malignancy, end-stage renal disease, or cirrhosis (*n* = 85), recent acute myocardial infarction or acute coronary syndrome within 30 days (*n* = 42), pre-existing severe heart failure with LVEF < 40% unrelated to COVID-19 (*n* = 56), non-COVID-19 acute myocarditis (*n* = 7), and incomplete clinical or laboratory data (*n* = 711). A final cohort of 314 patients with complete data was included in the analysis. Additionally, patients were classified into two groups based on the World Health Organization (WHO) COVID-19 severity classification criteria: mild/moderate group (*n* = 168); severe/critical group (*n* = 146).

### 3.2. Correlation Between Plasma sST2 Levels with Clinical Parameters

**Figure 2:**
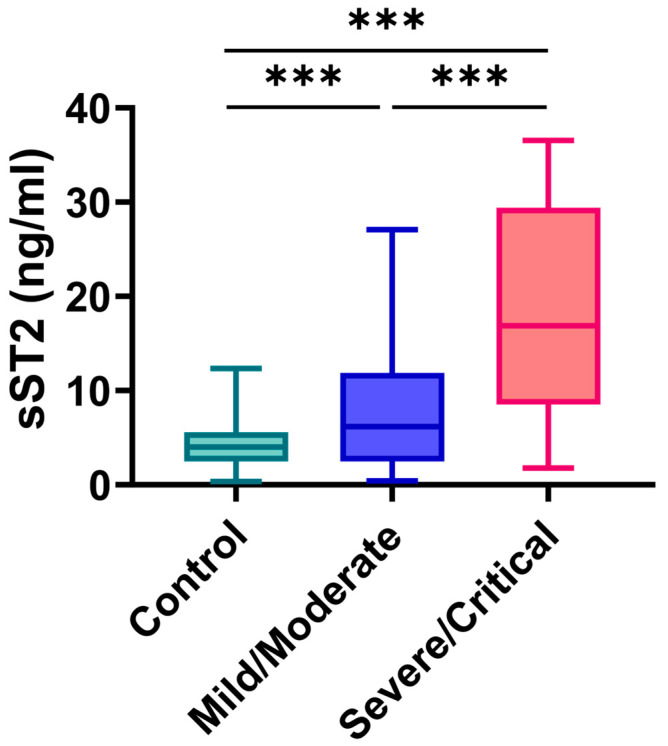
Plasma soluble ST2 (sST2) concentrations in mild/moderate and severe/critical COVID-19 patients. Plasma sST2 levels in patients with severe/critical COVID-19 (*n* = 146), mild/moderate COVID-19 (*n* = 168), and healthy controls (*n* = 213) were measured by ELISA. Data are presented as medians with interquartile ranges [Q_1_–Q_3_]; Kruskal–Wallis test was used to elevate the differences, *** *p* < 0.001.

**Figure 3:**
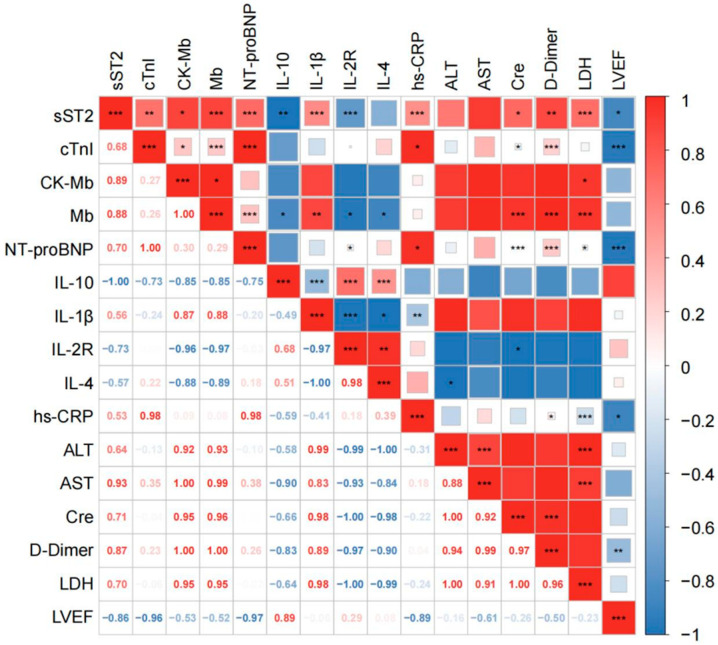
Correlation matrix of plasma sST2 and other clinical biomarkers in hospitalized COVID-19 patients. Spearman correlation analysis of plasma sST2 concentrations with a panel of cardiac injury biomarkers (cTnI, CK-Mb, Mb, and NT-proBNP), inflammatory cytokines (IL-10, IL-1β, IL-2R, IL-4, and hsCRP), and biochemical parameters (ALT, AST, creatinine, LDH, and D-dimer), as well as the left ventricular ejection fraction (LVEF). The color scale represents the strength and direction of correlations, with red indicating positive and blue indicating negative associations. The intensity of the color corresponds to the magnitude of the correlation coefficient (r). * *p* < 0.05, ** *p* < 0.01, and *** *p* < 0.001.

Plasma sST2 levels were significantly elevated in severe/critical COVID-19 patients [16.877 (8.507, 29.412) ng/mL], compared to those with mild/moderate disease [6.189 (2.499, 11.879) ng/mL] (*p* < 0.001), and were significantly higher than in healthy controls [4.003 (2.496–5.579) ng/mL] (*p* < 0.001) (Figure 2). To further investigate the relationship between sST2 levels and key clinical parameters, correlation analyses were performed (Figure 3). sST2 exhibited strong positive correlations with multiple cardiac injury markers, including cTnI (r = 0.68, *p* < 0.01), CK-Mb (r = 0.89, *p* < 0.05), Mb (r = 0.88, *p* < 0.001), and NT-proBNP (r = 0.70, *p* < 0.001), indicating a close association between sST2 and myocardial damage. Approximately 60% of severe cases had cTnI levels exceeding the 99th percentile cutoff, and nearly all had elevated NT-proBNP according to age-adjusted thresholds. Patients exceeding these clinical thresholds had significantly higher sST2 levels (median ~15 ng/mL vs. ~6 ng/mL; *p* < 0.001), indicating a strong association between elevated biomarkers and increased cardiovascular risk.

Furthermore, sST2 was inversely correlated with LVEF (r = −0.86, *p* < 0.05), suggesting that higher sST2 levels were associated with a greater impairment of cardiac function. Additionally, sST2 exhibited strong positive correlations with IL-1β (r = 0.56, *p* < 0.001), hs-CRP (r = 0.53, *p* < 0.001), and D-dimer (r = 0.87, *p* < 0.01), suggesting potential links between sST2, systemic inflammation, and coagulation dysfunction.

### 3.3. Plasma sST2 as an Independent Predictor of Cardiovascular Events and Mortality

To further evaluate the prognostic significance of sST2, we performed Cox proportional hazards regression analyses to examine the association between sST2 levels and the risk of cardiovascular events and all-cause mortality. In the univariate analysis, sST2, cTnI, Mb, NT-proBNP, IL-1β, hsCRP, and LDH were significantly associated with an increased risk of cardiovascular events (*p* < 0.05), whereas LVEF was inversely associated with cardiovascular event risks (HR = 0.941, 95% CI: 0.921–0.961, *p* < 0.001). In the multivariate Cox regression model, sST2 remained an independent predictor of cardiovascular events after adjusting for potential confounders (HR = 2.972, 95% CI: 1.174–7.521, and *p* = 0.022) (Table 2).

**Figure 4:**
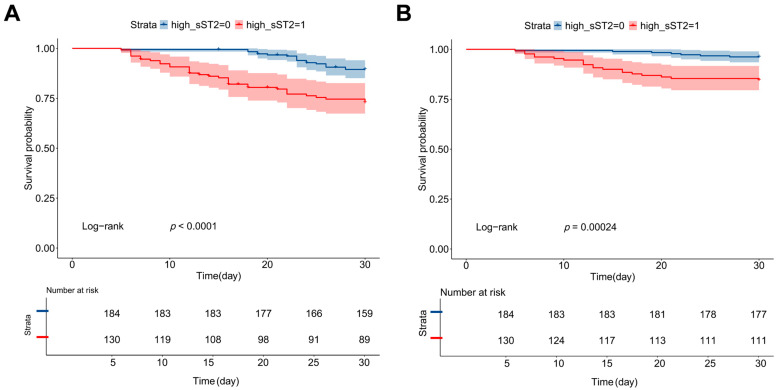
Kaplan–Meier analysis of cardiovascular events and all-cause mortality stratified by plasma sST2 levels in hospitalized COVID-19 patients. Patients were stratified into high and low sST2 groups based on the median plasma sST2 concentration at admission. Kaplan–Meier curve for the cumulative incidence of cardiovascular events (**A**) and all-cause mortality (**B**) in hospitalized COVID-19 patients. Shaded areas represent 95% confidence intervals.

Similarly, when all-cause mortality was analyzed as the primary outcome, both univariate and multivariate Cox regression models identified sST2 as an independent predictor of mortality (HR = 4.681, 95% CI: 1.346–16.280, and *p* = 0.015) (Table 2). The Kaplan–Meier survival analysis (Figure 4) further demonstrated that patients with higher plasma sST2 levels exhibited significantly reduced survival rates and a higher incidence of cardiovascular events compared to those with lower sST2 levels (log-rank *p* < 0.001).

### 3.4. Diagnostic Performance of sST2 in Predicting Cardiovascular Events and Mortality

To assess the diagnostic accuracy of sST2 in predicting cardiovascular events and all-cause mortality, a ROC curve analysis was performed. The performance of sST2 was compared with established cardiac injury biomarkers (cTnI, NT-proBNP).

**Figure 5:**
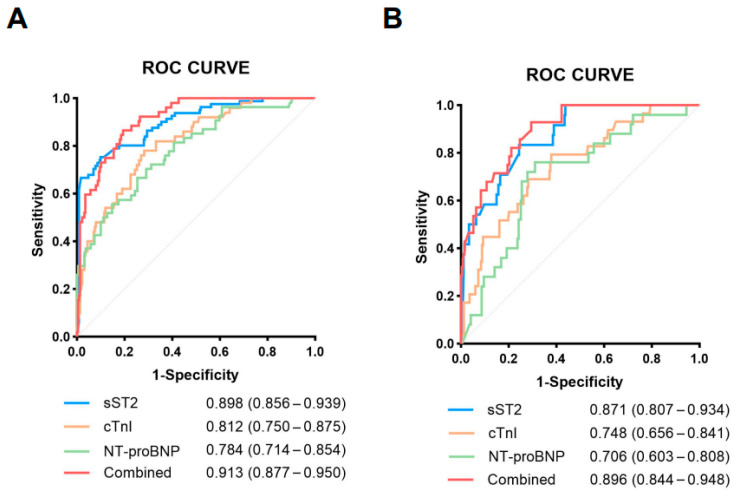
Receiver operating characteristic (ROC) curve analysis of sST2 and conventional biomarkers for predicting cardiovascular events and all-cause mortality in COVID-19 patients. Receiver operating characteristic (ROC) curves of plasma sST2, cTnI, NT-proBNP, IL-1β, and IL-2R for cardiovascular events (**A**) and all-cause mortality (**B**). AUC values for each biomarker are presented in the legend within each panel.

For predicting cardiovascular events, sST2 exhibited the highest diagnostic performance, with an AUC of 0.898 (95% CI: 0.856–0.939) (Figure 5A). The optimal cutoff value for sST2 was 20.691 ng/mL, demonstrating superior sensitivity and specificity. Among other biomarkers, cTnI (AUC = 0.812, 95% CI: 0.750–0.875) and NT-proBNP (AUC = 0.784, 95% CI: 0.714–0.854) showed a moderate predictive ability but remained inferior to sST2. Notably, the difference in AUC between sST2 and each of these markers was statistically significant (*p* = 0.018 vs. cTnI; *p* = 0.004 vs. NT-proBNP, DeLong’s test), confirming the added predictive value of sST2. When sST2 was combined with cTnI and NT-proBNP, the diagnostic performance improved, achieving an AUC of 0.913 (95% CI: 0.877–0.950), demonstrating enhanced predictive accuracy.

For predicting all-cause mortality, sST2 maintained the highest predictive accuracy, with an AUC of 0.871 (95% CI: 0.807–0.934) and an optimal cutoff value of 18.885 ng/mL (Figure 5B). While cTnI (AUC = 0.748, 95% CI: 0.656–0.841) and NT-proBNP (AUC = 0.706, 95% CI: 0.603– 0.808) exhibited a strong predictive ability, their performance remained slightly inferior to that of sST2. The difference in AUC between sST2 and cTnI was statistically significant (*p* = 0.035, DeLong’s test), as was the difference between sST2 and NT-proBNP (*p* = 0.012, DeLong’s test), confirming the superior predictive performance of sST2 for mortality. Similarly, when combined with sST2, cTnI and NT-proBNP further improved diagnostic performance, reaching an AUC of 0.918 (95% CI: 0.887–0.948).

Moreover, a sex-specific analysis revealed higher baseline sST2 levels in males (median ~7.2 ng/mL mild, ~18.5 ng/mL severe) versus females (median ~5.5 ng/mL mild, ~15.0 ng/mL severe). However, a ROC analysis showed comparable AUC values between sexes for events (0.90 men vs. 0.86 women, *p* = 0.45) and mortality (0.84 men vs. 0.82 women, *p* = 0.70). Optimal sex-specific cutoffs were ~19 ng/mL (men) and ~15 ng/mL (women), slightly improving stratification.

These findings indicate that sST2 is a reliable biomarker for predicting both cardiovascular events and mortality in COVID-19 patients, demonstrating a higher predictive accuracy than conventional cardiac biomarkers and inflammatory markers. Furthermore, the combination of sST2, cTnI, and NT-proBNP provides an even more robust diagnostic tool.

## 4. Discussion

This study provides evidence that plasma sST2 is significantly associated with myocardial injury, systemic inflammation, and coagulation dysfunction in hospitalized COVID-19 patients. We observed that sST2 levels were markedly elevated in severe/critical cases compared to mild/moderate cases. Higher sST2 concentrations independently predicted cardiovascular events and all-cause mortality, demonstrating a superior prognostic accuracy over conventional cardiac injury biomarkers. These findings underscore the potential of sST2 as a valuable prognostic biomarker for risk stratification in COVID-19 patients, facilitating the early identification of high-risk individuals and informing clinical decision-making.

Our study demonstrated strong correlations between plasma sST2 levels and key cardiac injury biomarkers, including cTnI, CK-Mb, Mb, and NT-proBNP, suggesting that sST2 is a reliable marker of myocardial injury in COVID-19 patients. This aligns with previous evidence indicating that COVID-19 can cause direct and indirect cardiac damage, often manifesting as acute myocardial injury, myocarditis, or the exacerbation of pre-existing cardiac disease [16,17]. The strong association between sST2 and reduced LVEF further reinforces its clinical relevance in assessing myocardial dysfunction in COVID-19. The IL-33/ST2 signaling axis plays a pivotal role in cardiac homeostasis. IL-33 binds to ST2L (membrane-bound ST2), exerting cardioprotective effects by attenuating cardiac fibrosis, hypertrophy, and apoptosis [18,19]. However, in pathological conditions such as severe COVID-19, elevated sST2 acts as a decoy receptor, neutralizing IL-33 and disrupting its protective functions, thereby promoting myocardial damage, fibrosis, and ventricular dysfunction [20]. Furthermore, systemic inflammation and hypoxia-related myocardial stress induced by severe COVID-19 may further elevate sST2 levels, compounding cardiac injury. Previous studies have established sST2 as a strong predictor of adverse cardiovascular outcomes in heart failure and acute coronary syndromes [11,21]. Our study extends these findings by demonstrating that sST2 is equally valuable in predicting myocardial injury in COVID-19 patients. Notably, unlike cTnI and NT-proBNP, which primarily reflect acute cardiac damage or volume overload, sST2 provides broader prognostic information related to cardiac remodeling and systemic inflammation, reinforcing its utility as a more comprehensive biomarker.

sST2 levels exhibited strong positive correlations with inflammatory cytokines, particularly IL-1β and hsCRP, suggesting that sST2 serves as a surrogate marker of excessive inflammatory activation in COVID-19. Given the role of a cytokine storm in severe COVID-19 cases, our findings highlight the potential role of sST2 in assessing disease severity and identifying patients at high risk for inflammatory complications. COVID-19 is characterized by a dysregulated immune response, leading to the excessive release of pro-inflammatory cytokines such as IL-1β, IL-6, and TNF-α, which contribute to tissue injury and multiorgan dysfunction [22,23,24]. sST2, as a decoy receptor for IL-33, amplifies this inflammatory cascade by blocking IL-33-mediated anti-inflammatory and cardioprotective effects [25]. Additionally, the IL-1β and sST2 may exacerbate systemic inflammation by promoting macrophage activation and endothelial dysfunction, thereby increasing the risk of thrombosis and cardiovascular complications [26].

The Kaplan–Meier survival analysis and multivariate Cox regression revealed that higher sST2 levels were independently associated with an increased risk of cardiovascular events and all-cause mortality. This suggests that sST2 can serve as a clinically useful biomarker for early risk stratification in COVID-19 patients, allowing for targeted interventions to mitigate adverse outcomes. While prior studies have evaluated NT-proBNP and troponins as predictors of cardiovascular risk in COVID-19, our study demonstrates that sST2 provides superior predictive accuracy for cardiovascular events and mortality [27,28]. Notably, the ROC curve analysis confirmed that sST2 exhibited the highest AUC compared to cTnI and NT-proBNP, emphasizing its greater prognostic utility. Furthermore, elevated sST2 could inform clinical management decisions, prompting closer cardiac monitoring, early ICU transfer, or targeted cardioprotective interventions. The integration of sST2 with conventional markers like troponin and NT-proBNP (combined AUC ~0.91–0.92) may further enhance clinical decision-making [25]. Patients in the lowest decile of sST2 (<~2 ng/mL) had no cardiovascular events or mortality during hospitalization. This observation aligns with evidence from heart failure studies showing that very low sST2 values indicate a favorable prognosis [29]. These findings suggest that sST2 could be integrated into current risk assessment models to enhance prognostic accuracy in COVID-19.

Moreover, treatment regimens were not standardized due to the retrospective study design, and we did not formally analyze interactions between treatment and biomarker levels. This limitation should be considered when interpreting our findings. Future prospective studies should investigate these interactions further.

This study has several limitations. First, it was a single-center retrospective study, which inherently limits the ability to establish causal relationships between sST2 levels and cardiovascular outcomes. Retrospective analyses are also prone to selection bias, misclassification, and incomplete clinical data, which may confound the observed associations. These methodological constraints, together with the single-center setting, may restrict the generalizability and translational applicability of our findings. Second, although the sample size was adequate for statistical analysis, it may not fully account for unmeasured confounders such as pre-existing cardiovascular comorbidities, heterogeneity in treatment protocols, or medication use. Third, we did not include long-term follow-up data, preventing the assessment of the prognostic significance of sST2 beyond hospitalization. Furthermore, a notable limitation of our study is the use of the non-FDA-approved ELISA for sST2 quantification rather than the clinically validated PRESAGE^®^ ST2 assay. Although the kit used has been reported to offer good reproducibility and sensitivity in previous translational studies, this difference in assay type may affect comparability across studies and clinical implementation. Future research should prioritize prospective, multicenter validation studies with long-term follow-up to confirm the utility of sST2 in diverse patient populations and settings. Moreover, exploring whether sST2 can serve as a therapeutic target or guide intervention strategies in COVID-19-associated cardiovascular complications remains an important direction.

Taken together, this study identifies sST2 as a superior prognostic biomarker in COVID-19, demonstrating its independent predictive value for cardiovascular events and mortality while offering greater diagnostic accuracy than conventional cardiac and inflammatory markers, highlighting its potential for improving risk stratification and clinical management in COVID-19-associated cardiovascular complications.

## Figures and Tables

**Table 1:** Baseline Characteristics of COVID-19 Patients.

Variables M (Q_1_, Q_3_)/*n* (%)	Total (*n* = 314)	Mild/Moderate (*n* = 168)	Severe/Critical (*n* = 146)	Statistic	*p*
Age	64.000 (53.000, 75.000)	59.000 (48.000, 72.000)	70.000 (57.250, 79.000)	Z = −3.914	**<0.001**
Sex, male	202 (64.331)	99 (58.929)	103 (70.548)	χ^2^ = 4.596	**0.032**
cTnI, ng/L	15.400 (5.150, 100.500)	7.800 (2.800, 20.875)	45.400 (10.400, 349.700)	Z = −7.013	**<0.001**
CK-Mb, μg/L	1.200 (0.600, 2.775)	0.700 (0.400, 1.375)	1.750 (0.900, 4.475)	Z = −5.748	**<0.001**
Mb, μg/L	87.450 (43.825, 214.125)	55.100 (33.800, 106.850)	146.400 (60.850, 636.750)	Z = −4.986	**<0.001**
NT-proBNP, ng/L	1266.500 (366.250, 5416.250)	836.000 (191.250, 2350.250)	2355.000 (662.000, 8987.000)	Z = −4.607	**<0.001**
IL-10, ng/L	7.060 (5.000, 19.300)	5.500 (5.000, 11.200)	9.100 (5.000, 25.150)	Z = −1.889	0.059
IL-1β, ng/L	6.300 (5.000, 16.250)	5.000 (5.000, 9.600)	7.100 (5.000, 19.975)	Z = −2.800	**0.005**
IL-2, ng/L	3.640 (3.087, 5.350)	4.135 (3.473, 5.350)	3.420 (2.990, 5.350)	Z = −0.798	0.425
IL-2R, kIU/L	1092.000 (794.000, 1760.000)	937.000 (703.000, 1595.500)	1212.000 (865.750, 1819.000)	Z = −2.301	**0.021**
IL-4, ng/L	3.000 (2.605, 3.232)	3.000 (2.678, 3.550)	2.925 (2.480, 3.130)	Z = −0.772	0.440
WBC, ×10^9^/L	7.480 (4.680, 10.560)	6.220 (3.860, 8.130)	9.410 (6.380, 13.200)	Z = -6.338	**<0.001**
Lymphocytes, ×10^9^/L	0.730 (0.450, 1.310)	0.940 (0.570, 1.580)	0.600 (0.380, 0.990)	Z = −4.575	**<0.001**
Neutrophil, ×10^9^/L	5.360 (2.760, 8.350)	3.830 (2.320, 6.260)	7.290 (4.695, 10.970)	Z = −6.706	**<0.001**
Platelet, ×10^9^/L	193.000 (127.000, 259.000)	195.000 (116.000, 249.000)	191.500 (141.750, 270.250)	Z = −1.506	0.132
Hb, g/L	108.000 (87.000, 128.000)	107.000 (83.000, 127.000)	108.500 (92.000, 128.000)	Z = −0.661	0.509
hsCRP, mg/L	38.300 (9.975, 96.150)	27.550 (4.775, 66.275)	50.100 (26.725, 119.600)	Z = −4.565	**<0.001**
LDL, mmol/L	1.875 (1.400, 2.522)	1.860 (1.510, 2.650)	1.890 (1.325, 2.460)	Z = −0.386	0.700
HDL, mmol/L	0.840 (0.640, 1.020)	0.905 (0.758, 1.042)	0.770 (0.620, 1.018)	Z = −1.927	0.054
ALT, IU/L	20.000 (13.000, 36.000)	19.000 (13.000, 34.500)	21.000 (12.750, 38.000)	Z = −0.638	0.524
AST, IU/L	24.000 (18.000, 36.750)	23.000 (16.000, 32.750)	27.000 (19.000, 41.250)	Z = −3.123	**0.002**
Cre, µmol/L	79.000 (61.000, 113.000)	75.000 (60.250, 97.500)	83.000 (62.500, 126.500)	Z = −1.787	0.074
LDH, U/L	270.500 (205.000, 398.000)	219.500 (182.000, 301.500)	351.500 (251.250, 475.000)	Z = −6.843	**<0.001**
LVEF%	60.000 (58.000, 61.000)	60.000 (60.000, 62.000)	60.000 (58.000, 60.000)	Z = −2.274	**0.023**
PaCO_2_	37.800 (35.300, 44.200)	39.200 (36.300, 43.000)	37.200 (32.850, 44.275)	Z = −1.245	0.213
PaO_2_	79.500 (63.800, 105.350)	91.200 (78.000, 114.000)	66.650 (57.675, 94.525)	Z = −3.727	**<0.001**
Fever	203 (64.650)	101 (60.119)	102 (69.863)	χ^2^ = 3.245	0.072
Cough	207 (65.924)	99 (58.929)	108 (73.973)	χ^2^ = 7.870	**0.005**
Sputum	131 (41.720)	60 (35.714)	71 (48.630)	χ^2^ = 5.359	**0.021**
Shortness of breath	78 (24.841)	18 (10.714)	60 (41.096)	χ^2^ = 38.619	**<0.001**
Dyspnea	54 (17.252)	14 (8.333)	40 (27.586)	χ^2^ = 20.208	**<0.001**
Chest pain	14 (4.459)	9 (5.357)	5 (3.425)	χ^2^ = 0.685	0.408
Chest distress	88 (28.025)	36 (21.429)	52 (35.616)	χ^2^ = 7.795	**0.005**
Fatigue	115 (36.741)	60 (35.714)	55 (37.931)	χ^2^ = 0.165	0.685
Gastrointestinal Symptoms	115 (36.624)	47 (27.976)	68 (46.575)	χ^2^ = 11.642	**<0.001**
Hypertension	150 (47.771)	71 (42.262)	79 (54.110)	χ^2^ = 4.395	**0.036**
Diabetes	84 (26.752)	37 (22.024)	47 (32.192)	χ^2^ = 4.121	**0.042**
Hyperlipidemia	13 (4.140)	7 (4.167)	6 (4.110)	χ^2^ = 0.001	0.980
Chronic renal failure	58 (18.530)	18 (10.778)	40 (27.397)	χ^2^ = 14.251	**<0.001**
Chronic liver disease	60 (19.108)	29 (17.262)	31 (21.233)	χ^2^ = 0.797	0.372
Chronic lung disease	18 (5.732)	6 (3.571)	12 (8.219)	χ^2^ = 3.123	0.077
Solid organ transplantation	24 (7.643)	10 (5.952)	14 (9.589)	χ^2^ = 1.463	0.226
Obesity (BMI ≥ 30)	15 (4.81)	4 (2.41)	11 (7.53)	χ^2^ = 4.460	**0.035**
Imaging findings	252 (86.598)	133 (81.098)	119 (93.701)	χ^2^ = 9.796	**0.002**
Respiratory failure	104 (33.121)	0 (0.00)	104 (71.233)	χ^2^ = 178.937	**<0.001**
Shock	21 (7.420)	0 (0.00)	21 (16.667)	χ^2^ = 28.264	**<0.001**
Organ failure	26 (9.253)	0 (0.00)	26 (20.968)	χ^2^ = 36.276	**<0.001**
Cardiovascular events	53 (16.879)	13 (7.738)	40 (27.397)	χ^2^ = 21.518	**<0.001**
Death	27 (8.599)	0 (0.00)	27 (18.493)	χ^2^ = 33.991	**<0.001**

Z: Mann–Whitney test, χ^2^: Chi-square test, M: Median, Q_1_: 1st Quartile, Q_3_: 3rd Quartile. Bold values indicate statistical significance at *p* < 0.05.

**Table 2:** Univariate and multivariate Cox proportional hazards regression analysis.

Variables	Outcome for Cardiovascular Events										Outcome for All-Cause Mortality									
	Univariate Analysis					Multivariate Analysis					Univariate Analysis					Multivariate Analysis				
	β	S.E	Z	*p*	HR (95%CI)	β	S.E	Z	*p*	HR (95%CI)	β	S.E	Z	*p*	HR (95%CI)	β	S.E	Z	*p*	HR (95%CI)
sST2	1.263	0.232	5.434	**<0.001**	3.537 (2.243–5.578)	1.089	0.474	2.299	**0.022**	2.972 (1.174–7.521)	1.349	0.341	3.96	**<0.001**	3.852 (1.976–7.508)	1.543	0.636	2.427	**0.015**	4.681 (1.346–16.280)
cTnI	0.001	0.000	3.328	**<0.001**	1.001 (1.001–1.001)	0	0	1.481	0.139	1.000 (1.000–1.001)	0.000	0.000	1.004	0.316	1.000 (1.000–1.000)					
CK-Mb	0.004	0.010	0.429	0.668	1.004 (0.985–1.025)						0.015	0.007	2.027	**0.043**	1.015 (1.001–1.029)	0.023	0.018	1.246	0.213	1.023 (0.987–1.060)
Mb	0.001	0.000	3.593	**<0.001**	1.001 (1.001–1.002)	0	0.001	0.142	0.887	1.000 (0.999–1.001)	0.002	0.000	3.897	**<0.001**	1.002 (1.001–1.002)	0.001	0.001	1.671	0.095	1.001 (1.000–1.002)
NT-proBNP	0.001	0.000	3.743	**<0.001**	1.001 (1.001–1.001)	0	0	−1.27	0.204	1.000 (1.000–1.000)	0.001	0.000	2.484	**0.013**	1.001 (1.001–1.001)	0	0	−0.302	0.763	1.000 (1.000–1.000)
IL-1β	0.023	0.008	3.034	**0.002**	1.024 (1.008–1.039)	0.006	0.014	0.459	0.646	1.006 (0.979–1.034)	0.028	0.008	3.574	**<0.001**	1.028 (1.013–1.044)	0.013	0.012	1.063	0.288	1.013 (0.989–1.038)
IL-2R	−0.000	0.000	−0.328	0.743	1.000 (1.000–1.000)						0.000	0.000	0.189	0.850	1.000 (1.000–1.000)					
hsCRP	0.005	0.002	2.930	**0.003**	1.005 (1.002–1.008)	0	0.003	−0.061	0.951	1.000 (0.993–1.006)	0.008	0.002	3.790	**<0.001**	1.008 (1.004–1.012)	0	0.004	−0.061	0.951	1.000 (0.992–1.007)
LDH	0.001	0.000	2.179	**0.029**	1.001 (1.001–1.002)	−0.002	0.002	−1.014	0.311	0.998 (0.995–1.001)	0.002	0.001	2.821	**0.005**	1.002 (1.001–1.003)	−0.002	0.001	−1.068	0.286	0.998 (0.995–1.001)
LVEF	−0.061	0.011	−5.764	**<0.001**	0.941 (0.921–0.961)	−0.078	0.033	−2.391	**0.017**	0.925 (0.868–0.986)	−0.023	0.024	−0.926	0.354	0.978 (0.932–1.026)					

Bold values indicate statistical significance at *p* < 0.05.

## Data Availability

The data presented in this study are available on reasonable request from the corresponding author. The data are not publicly available due to privacy or ethical restrictions.
